# Determination of Membrane Protein Transporter Oligomerization in Native Tissue Using Spatial Fluorescence Intensity Fluctuation Analysis

**DOI:** 10.1371/journal.pone.0036215

**Published:** 2012-04-27

**Authors:** Mikhail Sergeev, Antoine G. Godin, Liyo Kao, Natalia Abuladze, Paul W. Wiseman, Ira Kurtz

**Affiliations:** 1 Department of Physics, McGill University, Montréal, Québec, Canada; 2 Department of Chemistry, McGill University, Montréal, Québec, Canada; 3 David Geffen School Medicine, University of California Los Angeles, Los Angeles, California, United States of America; 4 Brain Research Institute, University of California Los Angeles, Los Angeles, California, United States of America; Swiss Federal Institute of Technology Zurich, Switzerland

## Abstract

Membrane transporter proteins exist in a complex dynamic equilibrium between various oligomeric states that include monomers, dimers, dimer of dimers and higher order oligomers. Given their sub-optical microscopic resolution size, the oligomerization state of membrane transporters is difficult to quantify without requiring tissue disruption and indirect biochemical methods. Here we present the application of a fluorescence measurement technique which combines fluorescence image moment analysis and spatial intensity distribution analysis (SpIDA) to determine the oligomerization state of membrane proteins *in situ*. As a model system we analyzed the oligomeric state(s) of the electrogenic sodium bicarbonate cotransporter NBCe1-A in cultured cells and in rat kidney. The approaches that we describe offer for the first time the ability to investigate the oligomeric state of membrane transporter proteins in their native state.

## Introduction

Intermolecular and intramolecular interactions play a fundamental role in almost all biochemical reactions in the cell. Regarding membrane transporter proteins, dimerization and higher order oligomerization have been proposed as important factors that modulate their activity [1]. Understanding the complex intermolecular interactions that ultimately influence the function and behavior of cells necessitates the availability of reliable quantitative techniques that can directly measure the density of proteins in addition to their oligomerization states in cells.

Various experimental techniques have been developed to obtain the distribution and extent of molecular interactions within cell membranes. Quantitative information regarding membrane protein interactions have historically been mainly aquired with various well accepted methods including chemical cross-linking, radiation protein inactivation, analytical ultracentrifugation, reconstitution titration experiments and SDS-PAGE using the nondenaturing detergents [2]. Electron microscopy (EM) is an additional useful approach to obtain the distributions of specific proteins in cell membranes since it provides high spatial resolution of protein interactions in cells [3]. In principle, a two-dimensional distribution function can be obtained from acquired EM images. However, the approach has difficulties with artifacts introduced by sample fixation procedures since the techniques used to prepare membrane samples for transmission EM are very damaging. A single molecule microscopy based approach can be used to quantitatively determine local stoichiometries based on quantitative criteria for assigning a defined number of fluorophores to each observed individual fluorescence peak [4], [5]. Single molecule techniques were shown to be able to measure local stoichiometries beyond the diffraction limit which makes it a powerful tool to study co-associations in biomembranes. However, the approach is limited to low density systems in which individual fluorescence peaks can be separately resolved and imaged in order to track macromolecular complexes. Förster resonance energy transfer (FRET) can measure the distances between sites on macromolecules labeled with donor and acceptor fluorescent dyes, and is sensitive to spatial scales on the order of about 1–10 nm [6]. The technique is able to trace the dynamics of aggregation in the system, however, it is challenging to obtain quantitative information because of difficulties in interpreting the energy transfer efficiency in terms of two-dimensional distributions. Another difficulty is that the Förster distances (distance for 50% transfer efficiency) are about 3–6 nm, which makes analysis of distributions of larger aggregates impossible. Furthermore, FRET does not provide information about the actual size of protein interactions in terms of the total number of subunits.

A group of microscopy based techniques that involve statistical analysis of fluorescence intensity fluctuations has been developed to measure chemical kinetics, dynamic molecular transport and interaction of proteins *in vivo*. The most widely used, fluorescence correlation spectroscopy (FCS) [7], has proven particularly sensitive in interaction studies [8], [9], [10]. Spontaneous fluctuations in the states of the fluorophore (molecular occupation number, quantum yield, etc.) within a laser beam focus generate time variations in detected fluorescence. Correlation analysis of these fluorescence fluctuations provides quantitative information about different transitions in the system. FCS is sensitive to molecular number densities, which is why it is well suited to study macromolecular oligomerization in biological systems. Protein interaction at equilibrium generates larger magnitude fluctuations in fluorescence intensity if different numbers of subunits have different fluorescent yields. Even though FCS has proven successful for measuring oligomeric distributions of rapidly moving cell macromolecules which have beam residency times on a short time scale (from microseconds to seconds), it becomes more problematic for measurements of membrane proteins whose mass action kinetics are slower [11], [12]. The photon counting histogram (PCH) method [13] and the fluorescence-intensity distribution analysis (FIDA) [14], which rely on intensity histogram analysis of the distributions of detected fluorescence photons, were developed for measuring densities and resolving oligomerization states of fluorescently labeled proteins. These temporal domain techniques are able to distinguish molecular species in solution or in cells by differences in their molecular fluorescence yields [15]. Fluorescence cumulant analysis (FCA), which is related to the PCH method, was introduced to resolve heterogeneous mixtures of biological molecules that rapidly diffuse in solution [16]. With FCA, it is possible to characterize the molecular brightness together with the number of molecules per observation volume for each fluorescent species present. The number and brightness (N&B) technique was also developed which allowed measurements of the average number of molecules and brightness in each image pixel in a fluorescence microscopy image time series [17], [18].

All of the techniques mentioned above rely on the temporal fluorescent fluctuation domain, making it difficult to obtain the distribution of oligomerization states in either fixed cells or tissues. In this paper, we apply two spatial fluorescent fluctuation domain techniques - fluorescence image moment analysis [19], [20] and SpIDA [21], [22] to single microscopy images to measure the oligomeric state of a membrane transport protein in its native environment without requiring tissue disruption and the use of detergents or other biochemical approaches. As a model system, we have applied these techniques to study the oligomeric state of the electrogenic sodium bicarbonate cotransporter NBCe1, a member of the SLC4 bicarbonate transporter family [23]. The importance of SLC4 proteins in mammalian biology is highlighted by the diseases that result from natural mutations in humans and targeted disruption of the transporters in murine models [23], [24].

The 10 transporters in the SLC4 family differ in their Na^+^- and Cl^−^ dependence and in their electrical properties [23]. Of the Na^+^-driven SLC4 transporters, the structural properties of NBCe1 (specifically the NBCe1-A variant) have been most thoroughly studied. NBCe1-A is predominantly expressed in the kidney proximal tubule where it mediates the absorption of bicarbonate [25], [26], [27], [28], [29]. In addition to the proximal tubule, NBCe1-A is also expressed in the eye [30], [31], salivary gland [32], and nasal submucosal glands [33]. The NBCe1-A monomer is a ∼140 kDa glycoprotein containing 1035 amino acids and is composed of 14 transmembrane regions (TMs) [34], [35]. Both the extreme N- and C-termini of NBCe1-A are located in cytoplasm, with a large extracellular loop between transmembrane segment 5 and 6 containing two glycosylation sites [34], [36]. The oligomeric state of NBCe1-A has recently been studied using the nondenaturing detergent, perfluoro-octanoic (PFO) acid [37], [38]. In HEK293 expressing NBCe1-A heterologously, it was shown in cell lysates that the cotransporter exists as monomers, dimers, and higher order oligomers [37]. However, in employing *in vitro* biochemical techniques one can always justifiably raise concerns as to whether the findings are affected by the choice of detergents used. In addition, potential oxididation effects resulting in artifactual disufide bond formation can also modify the oligomerization results. Clearly it would be more optimal to address questions regarding the oligomeric state of membrane transport proteins *in situ*. In this study, we demonstrate for the first time, the successful use of fluorescence image moment analysis [19], [20] and SpIDA [21], [22] to measure the oligomerization state of NBCe1-A heterologously expressed in cultured mammalian cells, and in native rat kidney.

## Experiment

### Fluorescence image moment analysis

Moment analysis is based on the spatial fluctuations of the fluorescent intensity. It allows one to extract important oligomerization information from single images collected by optical microscopy such as confocal laser scanning microscopy (CLSM), spinning disk scanning microscopy (SDCM) or total internal reflection fluorescence (TIRF) microscopy. It has been previously demonstrated that the number densities and brightness ratios of a mixed population of oligomers with different quantal brightness values can be determined by analyzing higher-order moments of the spatial fluorescence intensity fluctuations from individual images for specific ranges of densities and particle brightness ratios [39].

**Figure 1:**
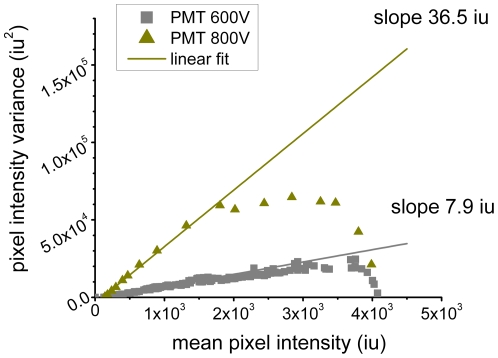
Characterization of the shot noise profiles for PMT detector. Plot of the variance as a function of the mean intensity for uniform illumination acquired with CLSM. Only the initial part of the data was taken into account for linear fitting.

We assume that all fluorescent species in our system are oligomers containing multiple subunits of the fluorescent label. We define an *n*-mer (monomer, dimer, etc.) as a fluorescent entity which *n* molecular subunits are in non random spatial proximity on a length scale below the width of the point-spread-function (PSF). The intensity brightness of a fluorescent oligomer (quantal brightness) is used to differentiate different oligomerization states. For example, a dimer with two fluorescent subunits emits on average twice as many photons as a monomer does, when quenching is negligible. For a single species, the amplitude of the image autocorrelation function is inversely proportional to the number of particles and the molecular brightness can be determined from the average intensity of the image divided by the number of particles. In the case of two populations present in the system, the overall integrated intensity will simply be the sum of the contributions from two oligomeric states:
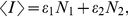
(1)where *N*_1_, *N*_2_ represent the number of oligomers per PSF defined beam area (BA) and 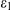, 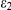 the quantal brightness (arbitrary intensity units, iu) of the first and the second populations respectively. The brightness ratio is defined as 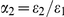. A series of normalized higher-order (*n* ^th^) moments of the spatial fluorescence intensity fluctuations within a single microscopy image are given by
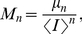
(2)where 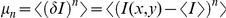 is the *n* ^th^ order intensity moment and 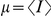. For fluorescently labeled NBCe1-A, multiple species are expected to be present ranging from the dim widely dispersed monomeric species up to higher order oligomeric, and hence brighter, populations. To resolve two populations and the relative brightness ratio 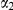, the first four moments are used. The contribution of the detector shot noise must be taken into account to correct the moments for the specific imaging conditions (see Figure 1). The detection limits and accuracy of fluorescence moment image analysis were characterized in detail using simulations and control experiments [39]. For multiple species analysis to work well, all population components need to contribute significantly to the spatial fluorescence intensity fluctuations.

### Spatial intensity distribution analysis

Our recently characterized SpIDA technique belongs to a family of histogram based methodologies [21], [22]. The technique is based on fitting super-Poissonian distributions of the histograms of fluorescence intensity calculated from single CLSM images. The values of fluorescent macromolecule densities and oligomerization states are obtained from the histogram fits.

The generalized intensity histogram for *N* particles inside BA can be calculated by weighting each density configuration with its proper probability assuming a Poisson distribution. is defined as the probality of measuring an intensity of *k* when having exactly *n* fluorescent particles with an average brightness ε of in the PSF. The fitting function becomes:
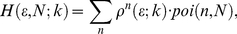
(3)where 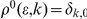. *H* is normalized over all intensity values so the integral over *k* yields unity. The histogram fitting function is calculated by computing the fluorescence intensity of all possible configurations of *n* particles in a given volume defined by the PSF. Each configuration is weighted according to their probability considering the Poisson spatial distribution of particles. This allows for the fit of an image intensity histogram to be performed. The fluorescent particle density, *N*, (particles per BA) and the quantal brightness, 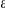, (intensity units per pixel dwell time, iu) are recovered, concurently, from the intensity histogram fit.

When two populations of fluorescent particles with distinct quantal brightness values are found randomly mixed in an image, the total histogram becomes the convolution of the two individual distributions:
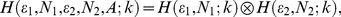
(4)where *N_i_* and 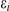 mean number of fluorescent particles per BA and quantal brightness for the *i*_th_ population and A is the total number of pixel used to generate the analyzed histogram. Histograms of fluorescence intensity acquired with analog photon multiplier tubes (PMTs) are broadened due to the shot noise and multiplication contributions. The broadening can be characterized based on the results of a control experiment (see Figure 1). The corresponding Matlab written software package for SpIDA is publicly available to the scientific community at http://www.neurophotonics.ca/en/tools/software.

Both fluorescence image moment analysis and SpIDA provide information about the interaction of the fluorescentlly labeled macromolecules. It has been shown that both independent fluorescence analysis techniques can accuratly resolve high protein concentration (>1000 µm^−2^) provided that the signal-to-noise ratio is sufficient [21], [22], [39]. Futhermore, since those two techniques are based on the spatial fluorescence intensity fluctuation and use single images as input data, the effects of photobleaching can be neglected. To properly apply both techniques, it is essential to establish an independent monomeric quantal brightness control and measure 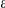. This ensures that if there are a mixture of monomers and oligomers present in the sample, fitting the data with a one-population model will yield a value of quantal brightness higher than 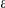 indicating that the monomer-oligomer model must to be used.

### Monolayer fluorescent dye sample preparation

Monomeric fluorescent dye samples were prepared by covalently attaching Alexa dyes to amine surface modified coverslips. The microscope coverslips (22×22 mm, No. 1.5; Electron Microscopy Sciences) were obtained from CEDARLANE Labs (Hornby, ON). The coverslips were cleaned for about 15 min in a Pyranha solution (1/3 (H_2_O_2_ 35%)+2/3 (H_2_SO_4_ 96%) mixture). The cleaned coverslips were stored in 14 MΩ milliQ water prior to experiments. Cleaned and air dried glass substrates were immersed into 5% 3-aminopropyltriethoxysilane (APTES) solution (Sigma-Aldrich, St. Louis, MO) at room temperature and gently shaken for about 30 min. Due to the observed degradation of the coating in air, the coverslips were kept in the solution until time of use. Alexa Fluor488 sulfodichlorophenyl (SDP) ester conjugate was obtained from Molecular Probes (Eugene, OR) to prepare the two-dimensional samples. The Alexa Fluor (488 and 647) SDP solution aliquots of variable concentrations were prepared in bicarbonate buffer (0.1 µM, pH 8.3) and then sonicated for 60 min before use. Next, 15 µL of Alexa Fluor 488 SDP solutions were applied to the coated coverslips and incubated for 5 min at room temperature followed by rinsing of the glass substrates with 95% lab grade ethanol. The obtained coating forms a stable peptide like bond which allowed for strong binding of the dye conjugate in a uniform manner on the glass coverslips. We assumed that APTES coated substrates, which provided free amine groups on the surface, should covalently bind only a monolayer of Alexa Fluor (488 and 647) sulfodichlorophenyl ester. The coverslips were then mounted on microscope slides for LSM imaging.

### Cell culture

CHO-K1 and HEK293 (catalog No. CRL-1573, ATCC) cell lines were cultured in Dulbecco's modified Eagle's medium, supplemented with 10% fetal bovine serum, 4 mM L-glutamine, 100 units/mL penicillin, 0.1 mg/mL streptomycin, 0.1 mM nonessential amino acids, and 0.5 mg/mL G418 to maintain transfection (Gibco, Carlsbad, CA). Cells were maintained in a humidified, 5.0% CO_2_ atmosphere at 37°C. Cells were transferred from the culture flask after detachment using 0.25% trypsin into petri dishes with a bottom coverslip insert (No. 1.5; MatTek, Ashland, MA) which were previously coated with 5 µg/mL fibronectin-like binding polymer (FNLP). Cells were grown in MatTek chambers to 80–90% confluency prior to transient transfections. The regular cell medium was replaced with OPTI-MEM (Invitrogen, Canada) one hour before transfection. Cells were transfected using various amounts (1 µg of DNA plasmid of interest and Lipofectamine LTX/PLUS reagent (catalog No. 15338–100; Invitrogen)) as described by the manufacturer. 2–4 hours post addition of the DNA, the plasmid solution was replaced with regular growth medium. The dishes were left in the incubator for another 12 hours to allow the cells express the protein. The cells were then fixed with 4% paraformaldehyde (PFA) for 10 min at RT conditions. Fixation the cells was followed by rinsing 3× with PBS and storage in 1 mL of PBS at 4°C until imaged.

### Cloning of NBCe1-A

The following human NBCe1-A contructs/plasmids were used in this study. 1) wt-NBCe1A in the pcDNA3.1 vector (Invitrogen); 2) NBCe1-A-EGFP: NBCe1-A was cloned into the pEGFP-C3 plasmid (Clontech). In this vector EGFP was coupled to the N-terminus of the cotransporter. 3) NBCe1-A-bungarotoxin binding peptide construct: The bungaroxin binding peptide sequence (WRYYESSLEPYPD; [40] was inserted into the large extracellular loop 3 of wt-NBCe1-A between residues Met-609 and Ser-610 using a site directed mutagenesis kit (Stratagene).

### Immunofluorescent labeling

CHO-K1 or HEK293 transiently expressing wt-NBCe1-A were incubated with −20°C methanol for 2 min. The cells were then rinsed extensively at RT with PBS. Cells were labeled for 60 min with a well-characterized primary rabbit anti-NBCe1-A antibody diluted 1∶500 [30]. After incubation with the primary antibody, the cell dishes were rinsed with PBS. Subsequentially, the cells were incubated with 1∶500 dilution of secondary mouse-anti-rabbit Alexa 647 antibody (Molecular Probes). Immunofluorescence staining was followed by rinsing the cells three times with PBS. The secondary antibody control samples (cells labeled with the secondary antibody only) were prepared along with the regular cell samples. Another set of control samples was prepared by immunostaining non-transfected cells with both primary and secondary antibodies. Similar studies were done in cells expressing NBCe1-A-EGFP except that the EGFP fluorescence was measured prior to methanol addition because of its quenching effect on EGFP fluorescence.

### Alpha-bungarotoxin labeling

Post fixation, cells transiently expressing the NBCe1-A-bungarotoxin binding peptide construct were rinsed 3× with PBS. Cells were then incubated with 1∶500 dilution of α-bungarotoxin Alexa Fluor 488 (Invitrogen) conjugate for 1 hour. The staining was followed by rinsing the cells three times with PBS. The nonspecific control samples (non-transfected cells labeled with α-bungarotoxin Alexa Fluor 488 conjugate) were prepared along with the regular cell samples.

### Rat kidney tissue samples

Rat kidney (catalog No. RF-901, Zyagen) was cut into thin slices that were immediately frozen in liquid nitrogen. 5 micron cryostat sections were attached to slides and stored at −80°C until used. The primary antibody against NBCe1-A (1∶100 in PBS) was applied for 40 minutes at room temperature and the slides were then washed thoroughly with PBS several times. Following several washes, goat-anti-rabbit IgG conjugated with Alexa 488 (1∶500 in PBS) was applied for 30 min at room temperature. The slides were washed with PBS, and mounted in Crystal/Mount (Biømeda Corp, Foster City CA).

### Confocal microscopy

The cell and tissue samples were imaged using an Olympus FluoView FV300 (Olympus America, Melville, NY) CLSM coupled to an Olympus IX71 inverted microscope equipped with a 60×1.4 NA oil immersion objective lens (Olympus PlanApo/IR). The eGFP and Alexa 488 samples were excited with a 40 mW multi-argon laser (458/488/515 nm, Melles Griot, Carlsbad, CA) using the 488 nm line. An Olympus FV-FCBGR dichromatic beamsplitter together with the emission filters BA510IF and BA530RIF (Chroma, Rockingham, VT) were used to efficiently reflect 488 nm wavelength and pass the emission wavelengths to the Channel 0. The Alexa 647 dye samples (Molecular Probes, Eugene, OR) and cells immunostained with Alexa 647 conjugate antibody were excited with a 10 mW 633 nm Helium-Neon laser (Melles Griot, Carlsbad, CA). The beam splitter DM630 together with the emission filter BA660IF were chosen to efficiently collect the Alexa 647 emission in the channel 1. The CLSM settings were kept constant for all samples and controls (selection of filters, dichroic mirrors, scan speed, pinhole and the step size for Z-stacks) so that valid comparisons could be made between measurements from different data sets. Acquisition parameters were set within the linear range of the PMT photon detector.

### Data analysis

For all images, the mean intensity of the background noise was calculated from empty dark regions in the images. To minimize the analysis biases caused by spatial heterogeneities in the spatial intensity distributions, we adjusted the sampling strategy to the features of a particular acquired image. MATLAB subroutines were written to calculate the *n* ^th^ order intensity moments of spatial fluorescence intensity fluctuations of the LSM images. The normalized moments of orders 2, 3 and 4 were calculated from the background corrected images and further corrected for the detector noise. The system of equations was then solved for the fluorescence yields and the population densities [39]. For SpIDA analysis, the intensity histograms were generated for a given background corrected image. To correct for the detector noise, each value in the histogram was substituted by a normalized Gaussian centered at the intensity *I* with the corresponding variance equal to 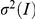 which was measured in the control calibration experiment (see Figure 1). The standard deviations of the recovered mean values for both types of analysis were obtained from the analysis of multiple simulated or LSM/TIRF images of the same sample type.

## Results

### PMT shot noise characterization

The ability of a CLSM to optically discriminate out-of-focus light makes it an ideal tool for basal membrane imaging of cells. However, with a PMT equipped CLSM, we do not directly measure the fluorescence photon counts, but detect the analog photoelectric current which is converted to an intensity. The moments experimentally measured from single CLSM images are not identical to those of the photon counts because of the shot noise contributions. The number density and brightness are strongly affected by this contribution. Therefore, it is necessary to take into account the shot noise and the detector noise in a separate control measurement. We placed a mirror in the focus of the microscope and measured the reflection signal which provided uniform illumination of the detector. The acquisition parameters were set to be constant for all samples and controls so that valid comparisons could be made between measurements from different data sets. From the acquired point scan recording for this control, we calculated the variance in the measured intensity time traces. The plot of the mean variance as a function of the mean intensity is shown in Figure 1. Only the initial part of the data were taken into account for linear fitting. The values of the slopes for this control are used in spatial fluorescence intensity fluctuation analysis applied to all CLSM image sets for the experiments presented in this work.

### Control measurement of the molecular brightness of monomeric EGFP

**Figure 2:**
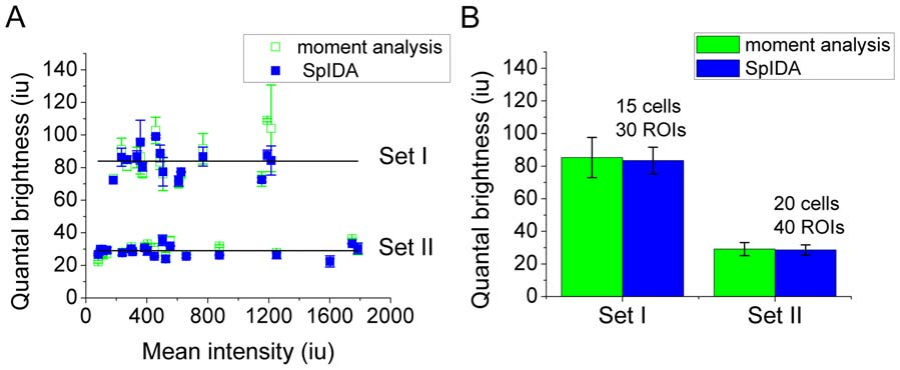
The measurements of quantal brightness of monomeric EGFP for two distinct sets of imaging conditions (referred as “Set I” and “Set II” throughout the text). Set I corresponds to pixel size of 0.046 µm, with the scan speed set to “fast” (18.1 µm/µs), and Set II - pixel size of 0.0921 µm, with the scan speed set to “slow” (10.1 µm/µs). The values of ε were recovered with both fluorescence moment image analysis and SpIDA for comparison purposes. The error bars were calculated as the standard error of images of multiple cells. A) measured quantal brightness for various values of the mean intensity; B) average values (with error bars) for sets I & II.

In order to carry out an independent measurement of the quantal brightness of monomeric EGFP, we transiently transfected CHO-K1 cells with monomeric EGFP (mEGFP) targeted to the membrane by attachment of a GPI moiety [41]. This version of GFP has been modified such that the probability of its oligomerization is minimized. The cells were chemically fixed and imaged with CLSM. The collected data sets were analyzed with fluorescence image moment analysis and SpIDA assuming one population of fluorescent entities was present. The average brightness from cells expressing monomeric EGFP was used as a control and the results were normalized to 1 EGFP monomeric equivalent unit (MEU). We collected multiple CLSM images of well adherent CHO-K1 cells transiently transfected with mEGFP with various expression levels of the mEGFP plasmid. We obtained the identical values of the monomeric quantal brightness (within statistical error) for the range of densities by two orders of magnitude. We acquired the images with two distinct sets of imaging conditions (referred as “Set I” and “Set II” throughout the text). Set I corresponds to pixel size of 0.046 µm, with the scan speed set to “fast” (18.1 µm/µs), and Set II - pixel size of 0.0921 µm, with the scan speed set to “slow” (10.1 µm/µs). The results of spatial fluorescence fluctuation analysis applied to the acquired image data sets is shown in Figure 2.

### Fluorescent dye measurements

Monolayer samples of monomeric fluorescent dye were prepared by covalently attaching Alexa Fluor SDP conjugates to amine surface modified coverslips. The protocol we employed would homogeneously distribute bound fluorescent dyes on the coverslip surface to produce samples with a 2D geometry. These samples were imaged with CLSM. No surface defects or gradients were observed in these monolayer coatings.

**Figure 3:**
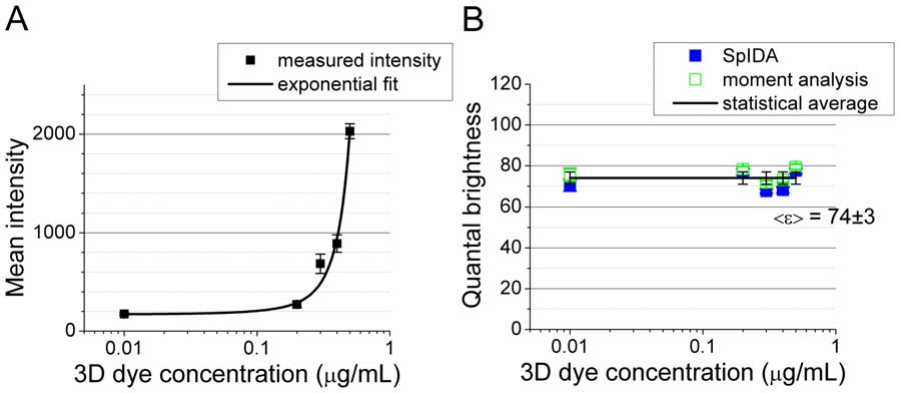
Quantal brightness of Alexa Fluor 488 measured for 2D samples prepared with different values of concentration in 3D solution. (A) Mean intensity measured from ROIs. (B) The values of the quantal brightness measured with fluorescent image moment analysis and SpIDA as a function of the dye concentration in solution. The error bars were calculated as the standard error of multiple images taken at different locations in the sample.

We experimentally confirmed that our protocol yielded coatings with a constant 2D surface density using fluorescent dye solutions of different concentrations. We applied one-population fluorescence moment image analysis and SpIDA to CLSM images of the samples with a wide range of concentration values (0.01–0.5 µg/mL in solution). We produced a calibration single exponential curve that relates 2D surface density on glass to bulk concentration in solution (see Figure 3A). The range of concentrations explored in the experiments was selected based on the CLSM detection limits and steric effects of the SDP Alexa dye conjugates. The resulting mean intensity values of CLSM images of samples prepared with concentration values lower than 0.005 µg/mL were below the dark count noise level. Based on the protocol we employed, samples prepared with dye concentrations higher than 1 µg/mL exhibited steric effects and resulted in quenching between neighboring fluorophores as measured via pixel intensities. According to the manufacturer, the ester molecules were labeled with single Alexa dyes. We applied fluorescence image moment analysis and SpIDA to CLSM data sets of Alexa Fluor 488 SDP monolayer samples with a wide range of surface density values (0.01–0.5 µg/mL) to obtain the values of the brightness (see Figure 3B). In the concentration range used for this assay, no significant quenching was observed (also presented in [21]).

### Determining the oligomeric state of NBCe1-A in cultured cells

**Figure 4:**
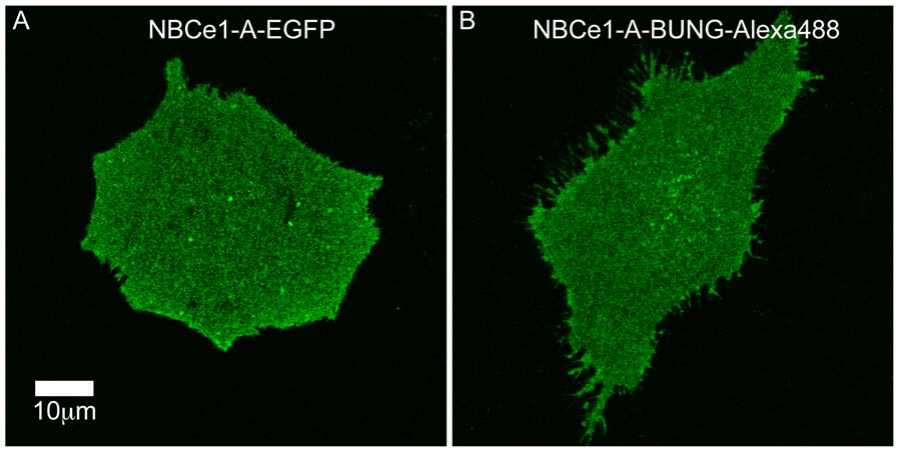
Sample CLSM images of CHO-K1 cells transiently transfected with NBCe1-A. (A) NBCe1-A-EGFP. (B) NBCe1-A-α-bungarotoxin mutant expressed in cells, and labeled with Alexa 488-α-bungarotoxin conjugate. In both cases, basal membranes of highly adherent cells was imaged. The cells were chemically fixed prior to imaging. The pixel size is 0.046 µm. The images were taken under the identical acquisition condition so that valid comparisons between different images could be made. The image contrast was enhanced in both panel A and B for visualization purposes.

**Figure 5:**
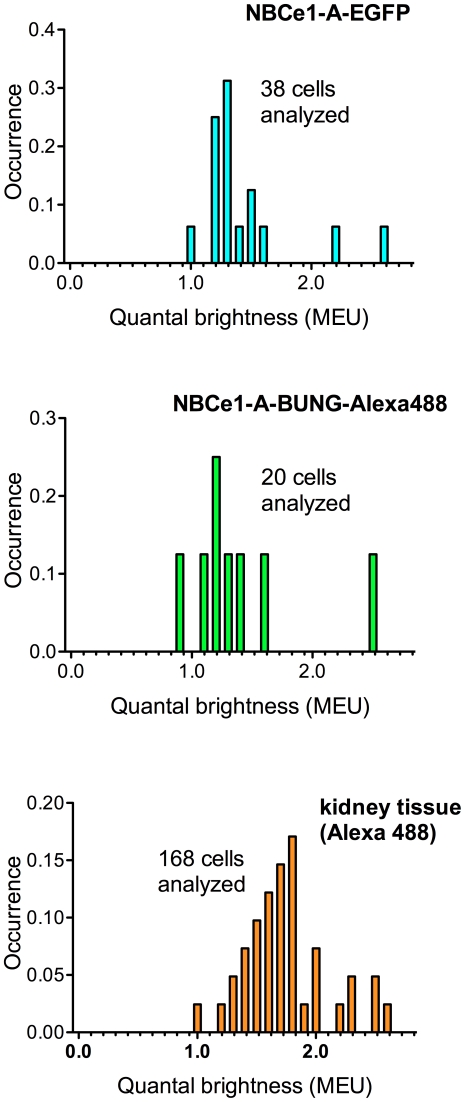
Spatial fluorescence intensity moment analysis of the NBCe1-A oligomerization states. The percent occurrence graphs (A–C) show the recovered values of quantal brightness normalized to MEU. The experiments were carried out on fixed cells. All of the measurements summarized in each histogram were carried under identical collection conditions.

CLSM images of adherent CHO-K1 cells transiently transfected with NBCe1-A-EGFP were collected (Figure 4A). Fluorescence image moment analysis was applied to the collected data. We used the values of the monomeric quantal brightness of EGFP obtained in the described control experiment (see Figure 2) to normalize the recovered values of the cotransporter's quantal brightness to 1 MEU. The results of oligomerization measurements of NBCe1-A-EGFP are shown in Figure 5A.

**Figure 6:**
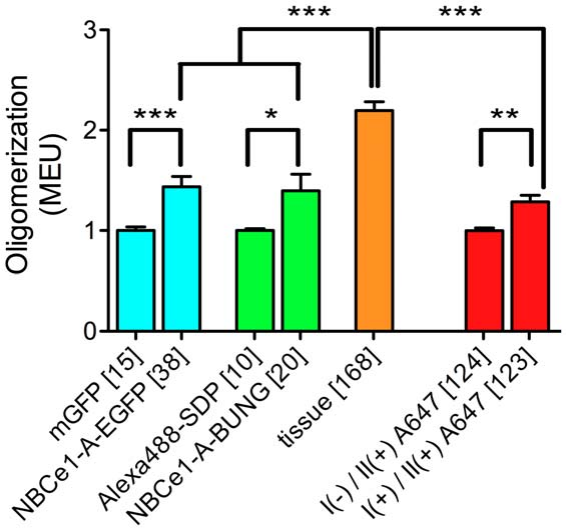
Spatial fluorescence intensity moment analysis of NBCe1-A oligomerization state in expression systems and native tissues. The cotransporter is predominantly a monomer when it is expressed heterologously in CHO-K1 cells, and is a dimer or rarely higher order oligomer in rat kidney tissue. The error bars represent the standard error of the means obtained from multiple cells. Numbers in square brackets represent *n* cells. Nonparametric *t*-tests were performed on the selected data sets (*** = p<0.001, ** = p<0.01, * = p<0.05). All of the measurements were carried out under identical collection conditions.

As an alternative approach, we used Alexa 488-α-bungarotoxin conjugate to label CHO-K1 cells transiently expressing the NBCe1-A-α-bungarotoxin binding mutant. A typical CLSM image is shown in Figure 4B. We assumed that the presence of α-bungarotoxin, to which Alexa 488 dye is conjugated, did not affect the quantal brightness of the dye. As a monomeric control for oligomerization measurement of NBCe1-A-α-bungarotoxin binding mutant labeled with Alexa-488-α-bungarotoxin, we measured the value of quantal brightness of Alexa 488 dye immobilized on glass cover slips as described (see Figure 3) which was normalized to 1 MEU and used for calibration purpose. The fluorescence image moment analysis revealed the distribution of quantal brightness values similar to that obtained for NBCe1-A-EGFP (Figure 5A, 5B). A non-parametric *t*-test was carried out to compare the brightness value distributions obtained for NBCe1-A-EGFP and NBCe1-A-α-bungarotoxin mutants and revealed that the two measurements in the heterologous expression system were not significantly different (Figure 6).

### In situ NBCe1-A oligomerization measurements in rat kidney

**Figure 7:**
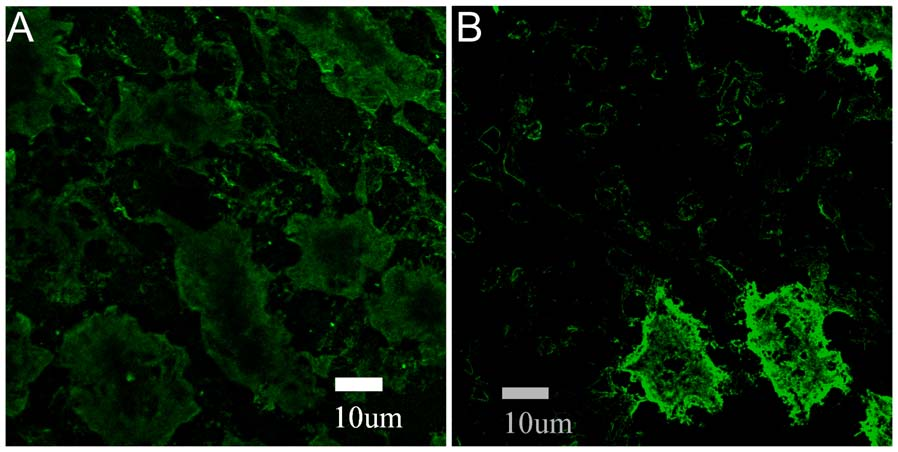
Sample CLSM images of native NBCe1-A on basolateral cell membranes of rat kidney. Post fixation, tissues were immunolabeled with the primary anti-wt-NBCe1-A antibody (I) and the secondary (II) Alexa Fluor 488 conjugated antibody. Panel A shows non-specific secondary antibody staining in the absence of the primary. Panel B shows kidney cells immunostained with both antibodies. The pixel size is 0.0921 µm. The images were taken at identical acquisition conditions so valid comparisons between different images could be made. The image contrast was enhanced for visualization and comparison purposes.

**Figure 8:**
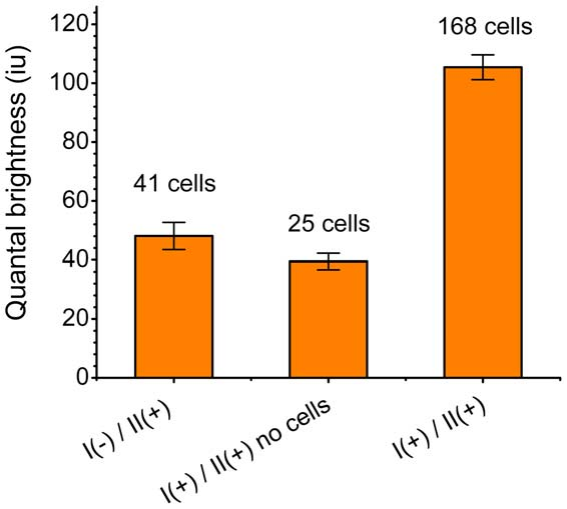
The fluorescence image moment analysis of the oligomerization state of the native NBCe1-A in rat kidney tissues. CLSM images of the native NBCe1-A immunostained with primary anti-wt-NBCe1-A antibody (I) and the secondary Alexa Fluor 488 conjugated antibody (II) were analyzed (the right bar). For establishing the monomeric label control, images of tissues immunostained with the secondary antibody in the absence of the primary were analyzed (the bar on the left). As an extra control, regions in between neighboring kidney cells (empty regions) in CLSM images of kidney tissues labeled with both anti-wt-NBCe1-A primary antibody and the fluorescently labeled (Alexa Fluor 488) secondary antibody were analyzed (the middle bar). The moment analysis reveals non-significant binding of both primary and secondary antibody together. The error bars represent the standard error of the mean obtained from multiple cells.

We applied fluorescence image moment analysis to CLSM images of homogenously distributed secondary antibody non-specifically bound on the surface of rat kidney cells. A typical CLSM image of native NBCe1-A on the cell basolateral membrane in rat kidney treated with the secondary (II) Alexa Fluor 488 conjugated antibody only is shown in Figure 7A. The measured values of quantal brightness are shown in Figure 8, left bar. We repeated the measurement on images of kidney labeled with both wt-NBCe1-A primary antibody (I) and the fluorescently labeled (Alexa 488) secondary antibody (II) by analyzing regions in between neighboring proximal tubule cells (empty regions). The analysis of homogenously distributed primary and secondary antibody within the intercellular regions yielded a value of the quantal brightness similar to that obtained for control images of homogenously distributed secondary antibody non-specifically bound on the basolateral aspect of rat kidney cells in the absence of the primary antibody (Figure 8, middle bar). Since the values of the quantal brightness were comparable (“I(−)/II(+)” vs “I(+)/II(+)no cells” in Figure 8), the nonspecific binding of the primary anti-wt-NBCe1-A antibody is negligible. This strongly suggests that the stoichiometry of secondary to primary is largely 1∶1. The average value of quantal brightness (44±4 iu) was used as the monomeric brightness. It was normalized to 1 MEU and used to calibrate the oligomerization measurement of immunostained NBCe1-A in native tissue. We then applied fluorescence image moment analysis to CLSM images of rat kidney labeled with both anti-wt-NBCe1-A and secondary (Alexa 488) antibody. A typical CLSM image of NBCe1-A immunolabeled with both the primary anti-wt-NBCe1-A antibody (I) and the secondary (II) Alexa Fluor 488 conjugated antibody is shown in Figure 7B. The corresponding occurrence plot shown in Figure 5C, bottom graph, suggests that the distribution of oligomers of the cotransporter is significantly different compared to that measured in heterologous expression systems. The moment analysis of CLSM images of immunolabeled NBCe1-A on proximal tubule basolateral cell membranes in rat kidney tissue yielded a quantal brightness value of 2.2±0.1 MEU (Figure 8, bar on the right).

**Figure 9:**
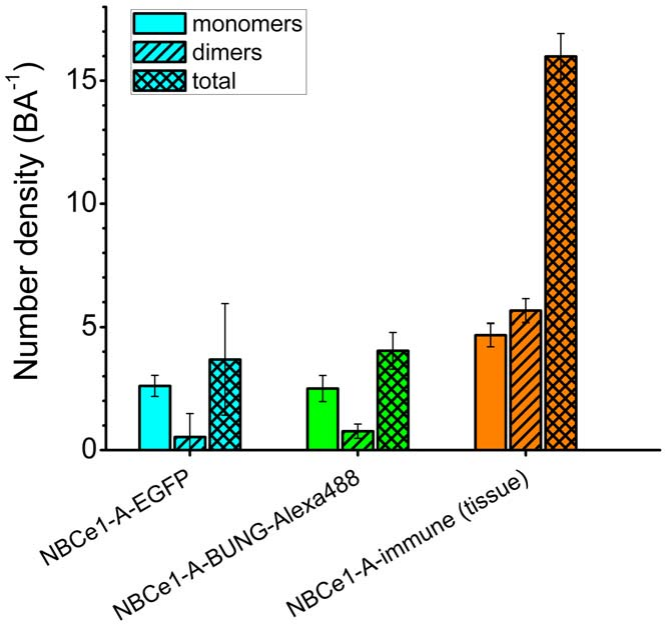
SpIDA measurements of NBCe1-A monomer-dimer density in expression systems (CHO-K1 cells) and native tissues. SpIDA was applied to CLSM images assuming a monomer-dimer mixture model (Equation 4). The values of monomeric, dimeric and total subunit number density were obtained for NBCe1-A in heterologous expression systems and tissues. The recovered number density of monomeric NBCe1-A in tissues is expected to be overestimated based on the presence of non-specific antibody binding. The error bars represent the standard error of the mean obtained from multiple cells. All of the measurements were carried out under identical collection conditions.

We applied two-population SpIDA to CLSM image sets of NBCe1-A-EGFP, NBCe1-A α-bungarotoxin-Alexa 488 and native immunolabeled NBCe1-A in rat kidney assuming that there is a distribution of monomers and dimers present in the images. Since all three utilized different fluorescent tags, the corresponding previously measured control values of the monomeric quantal brightness were used for calibration in the two-population SpIDA analysis. Figure 9 shows the recovered values of the monomer and dimer surface density of the cotransporter.

### Nonspecific antibody binding control

**Figure 10:**
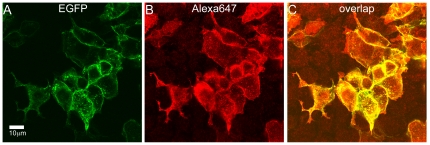
Dual-color CLSM images of HEK293 cells transiently expressing NBCe1-A-EGFP immunostained with Alexa Fluor 647 conjugated antibody. Post fixation, cells were immunolabeled with the primary anti-wt-NBCe1-A antibody and the secondary Alexa Fluor 647 conjugated antibody. Panel A shows the fluorescence image taken in channel 0 (green), Panel B shows the image recorded in channel 1 (red), and Panel C is the overlap of the two. The image contrast was enhanced for better visualization of non-specific binding of the secondary Alexa Fluor 647 conjugated antibody to HEK293 cells. The pixel size is 0.0921 µm.

**Figure 11:**
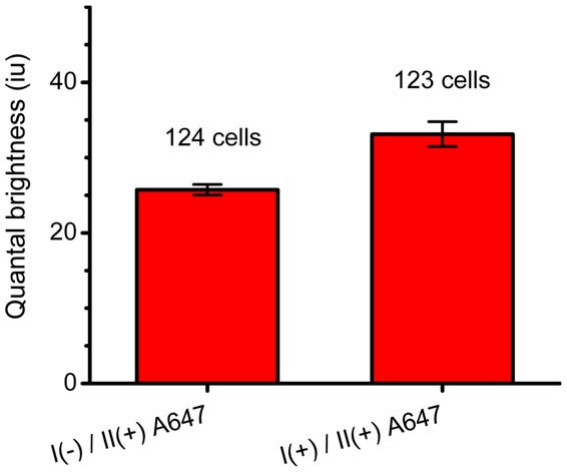
Non specific binding control of the antibody used for kidney tissue staining. HEK293 cells were transiently transfected with NBCe1-A-EGFP mutant. Post fixation, cells were immunolabeled with both primary anti-wt-NBCe1-A antibody (I) and secondary Alexa Fluor 647 conjugated antibody (II) (right bar), and with secondary Alexa Fluor 647 conjugated antibody alone (left bar). The moment analysis reveals non-significant binding of both primary and secondary antibody together. The secondary antibody was identical to the one used for tissue immunostaining except it was labeled with Alexa 647.

HEK293 cells were transiently transfected with EGFP-tagged NBCe1-A construct allowing us to analyze images of only those cells which were expressing NBCe1-A-eGFP. As with CHO-K1 cells, the HEK293 cells do not express endogenous NBCe1-A allowing us to conduct transfections of various NBCe1-A mutants and study their oligomerization distributions systematically without complications from endogenous protein. HEK293 cells were immunostained with the same primary anti-wt-NBCe1-A antibody which previously was used for rat kidney. The secondary antibody was also identical to that used for tissue staining, however, we chose Alexa 647 to be the fluorescent dye tagging the antibody (in contrast to Alexa 488 used for tissue staining) to allow dual-color CLSM imaging of both EGFP and Alexa 647 (see Figure 10). The overlap image of EGFP and Alexa 647 clearly shows the cells expressing NBCe1-A-EGFP (the image overlay results in a yellow color in regions of colocalization). Cells which do not express the cotransporter are observed in channel 1 (red) since the fluorescence signal comes only from the non-specific secondary antibody tagged with Alexa 647. We then applied fluorescence image moment analysis to CLSM images of homogenously distributed secondary antibody non-specifically bound on the surface of HEK293 cells. The measured value of quantal brightness (shown in Figure 11, the bar on the left) was used as the monomeric brightness calibration and was normalized to 1 MEU. We then applied fluorescence image moment analysis to CLSM images of HEK293 cells expressing NBCe1-A-EGFP immunolabeled with both primary anti-wt-NBCe1-A and secondary Alexa 647 tagged antibodies. The image analysis yielded a quantal brightness value of 1.3±0.1 MEU (compared to 2.2±0.1 MEU in tissue) (Figure 11, the bar on the right).

## Discussion

The ability to quantify the density of proteins together with their oligomerization states is required for the study of complex molecular interactions *in situ*. Various experimental methods, based on resonance energy transfer and temporal fluorescence fluctuation spectroscopy, have been developed for this purpose [6], [7], [10], [17], [18]. Although proven to be useful, these methods are unable to provide information regarding the oligomerization state of proteins in native tissue samples.

In this study we have demonstrated for the first time the application of a novel methodology, fluorescence image moment analysis with SpIDA, for determining the oligomeric state of membrane transporters *in situ* using standard fluorescence microscopy without requiring tissue disruption and subsequent biochemical approaches. We applied these techniques to study the oligomeric state of the electrogenic sodium bicarbonate cotransporter NBCe1-A as a model system. Our results show for the first time in the native kidney that NBCe1-A is dimeric.

The oligomerization measurements carried out in this work first required an independent “yardstick” for monomeric quantal brightness. Based on the results of the independent measurements of the quantal brightness of mEGFP we first used the average brightness from cultured cells expressing mEGFP as a control and normalized the obtained results to 1 MEU. To explore how the expression level (surface density of fluorophores) affected the measurements of molecular brightness of EGFP, we applied the spatial fluorescence fluctuation analysis to the collected multiple CLSM images of well adherent CHO-K1 cells transiently transfected with mEGFP with various expression levels of the mEGFP plasmid. Significant changes in the mean intensity (two orders of magnitude in surface density) did not affect the values of the measured quantal brightness of mEGFP which confirms that the vast majority of the mEGFP were, indeed, monomeric. The average values of molecular brightness obtained for Set I and Set II were subsequently used as monomeric controls for the oligomerization measurements of NBCe1-A-EGFP.

As an alternative approach, we used an Alexa Fluor 488 for α-bungarotoxin fluorescent labeling of NBCe1-A. Similar to the mEGFP brightness control data, we measured the monomeric quantal brightness of Alexa Fluor 488 dye immobilized on cover slips. The mean intensity and molecular brightness values obtained using spatial fluorescence fluctuation analysis applied to the collected data sets showed that both techniques provided identical values of the brightness for the wide range of surface densities. Even though we observed a significant change in the mean intensity of the collected images over the range of surface density values, the measured quantal brightness did not exhibit any dependence on the surface density. Our findings indicate that monolayer preparations can be used for calibrating the monomeric quantal brightness provided that the molecules that are labeled fluorescently do not alter their quantal brightness. Similarly, our previous study showed that, for a wide protein concentration range, in image time series where significant photobleaching was observed (high laser power used), the resolved quantal brightness remained constant over time while the recovered density was shown to be decreasing exponentially [21]. The average brightness value of Alexa 488 dye obtained from the spatial fluctuation analysis of CLSM images obtained was normalized to 1 MEU and this calibration control was used for α-bungarotoxin labeling of NBCe1-A. As observed for the measurement of the monomeric brightness of mEGFP, significant changes in the surface density of Alexa Fluor 488 dye did not affect the values of the measured quantal brightness.

We intially examined basal membranes of highly adherent CHO-K1 cells expressing EGFP-tagged NBCe1-A. CHO-K1 cells were chosen for the transfection experiments because of their large flat surface area and ability to strongly adhere. CLSM images of cells transiently transfected with NBCe1-A-EGFP displayed mainly a plasma membrane distribution. The significant increase in the brightness of NBCe1-A-EGFP compared to that of mEGFP suggested the coexistence of monomers and dimers based on the respective magnitudes. Similar to NBCe1-A-EGFP, images of cotransporter labeled with Alexa-488-α-bungarotoxin displayed mainly a plasma membrane distribution. In our constructs, EGFP was coupled to the N-terminus of the cotransporter while the bungaroxin binding peptide sequence was inserted into the large extracellular loop 3 of the wild type NBCe1-A. Since the fluorescence image moment analysis revealed the distribution of quantal brightness values similar to that obtained for NBCe1-A-EGFP, we conclude that the choice of NBCe1-A construct and the fluorescent probe it was tagged with or the choice of the tag insertion position, do not affect the measured value of the oligomerization state of the cotransporter in this cell expression system. The data indicate that NBCe1-A is both monomeric and dimeric when transiently expressed in CHO-K1 cells. Moreover, the oligomerization distribution of NBCe1-A-EGFP and the cotransporter mutant labeled with Alexa-488-α-bungarotoxin followed the same trend which validates our assay for the monomeric controls.

Heterologous expression systems provide a great deal of flexibility for studying oligomerization states of proteins in a well defined sytem. However, the heterologous expression of a protein could potentially affect its oligomeric state because of the fact that it is in a non-native environment. Therefore we extended our analysis to assess the oligomerization state of NBCe1-A in its native environment (the kidney proximal tubule) for the first time. We applied fluorescence image moment analysis to CLSM images of immunofluorescently labeled endogenous NBCe1-A in rat proximal tubules. The values of quantal brightness measured from CLSM images of homogenously distributed primary plus fluorescent secondary antibody within the intertubular regions along with the control images of homogenously distributed fluorescent secondary antibody non-specifically bound the basolateral aspect of rat kidney cells (in the absence of the primary antibody) were used as monomeric brightness controls and were normalized to 1 MEU for calibration.

The results of the moment analysis of CLSM images of immunolabeled NBCe1-A in rat kidney proximal tubule basolateral cell membranes suggested that the distribution of the oligomeric state of the cotransporter differes from that measured in heterologous expression systems. Even in the presence of non-specific secondary antibody binding which strongly affects the monomeric population, the significant increase in the measured quantal brightness indicated the presence of a dominant population of dimers. SpIDA was then used to resolve monomer-dimer distributions of fluorescently tagged NBCe1-A in CLSM images of intact cells and rat kidney. The monomer-dimer SpIDA data confirmed that NBCe1-A is predominantly a monomer and rarely a dimer when transiently expressed in CHO-K1 cells. The predominant population of dimeric native NBCe1-A was shown by SpIDA in kidney tissue. The recovered number density of dimeric NBCe1-A is expected to be underestimated based on a typical low antibody affinity. Since non-specific antibody binding is expected to be monomeric by nature (provided that the concentration is kept at its minimum for immunolabeling), we expect the number density of monomeric native NBCe1-A obtained with two-population SpIDA to be overestimated (due to the presence of non-specific label) leaving the value of the dimeric density unaffected.

Our results show that native NBCe1-A is predominantly a dimer in rat kidney. The detection limit of the image analysis does not allow us to determine the exact number density values for higher order oligomers, however, we conclude that their presence is insignificant based on the results of the fluorescence image moment analysis and the two-population SpIDA. In addition, to address the validity of the oligomerization measurement of native NBCe1-A in rat kidney, we also carried out an independent control for non-specific binding of the antibody used for kidney staining and showed that the measurements of the oligomerization state of native NBCe1-A in rat kidney were not biased by potential binding of multiple antibodies to the cotransporter.

Our immunofluorescent study in HEK293 cells is important for multiple reasons. First, it confirms that the results obtained for the rat kidney tissue are not caused by an artefact of the labeling itself. Second, it shows that the vast majority of the NBCe1-A transporters are labeled with a complex of a single primary antibody and a single fluorescently labeled secondary antibody, and that the difference between the expression systems and *in situ* does not correspond to a choice of a single expression system. Oligomerization states of the NBCe1-A transporters were obtained for two expression systems (CHO-K1 and HEK293) and three different types of labeling (EGFP, single Alexa 488 and immunofluorescence) without significant difference emphazing that the results are unbiased. Conversely, significantly different results were obtained for native animal tissue compared to the expression sytems while the labeling approach remained the same.

In conclusion, spatial fluorescence fluctuation analysis can be used in the native tissue environment, where one can address for the first time questions that involve the time dependence, spatial compartmentation, and regulation of membrane protein transporter oligomerization without requiring tissue disruption. Since oligomerization is an important aspect for understanding the structure-function properties of membrane transporters, fluorescence image moment analysis coupled with SpIDA will become an important new biological tool for addressing these questions directly in cells and tissues.
